# Immune cells and checkpoints in pancreatic adenocarcinoma: Association with clinical and pathological characteristics

**DOI:** 10.1371/journal.pone.0305648

**Published:** 2024-07-02

**Authors:** Maria Auxiliadora de Paula Carneiro Cysneiros, Magno Belém Cirqueira, Lucas de Figueiredo Barbosa, Ênio Chaves de Oliveira, Lucio Kenny Morais, Isabela Jubé Wastowski, Vitor Gonçalves Floriano

**Affiliations:** 1 Diagnostic and Therapeutic Support Division of Clinical Hospital, Federal University of Goias, Goiania, Brazil; 2 Pathology Department, Federal University of São Paulo, São Paulo, Brazil; 3 Surgery Department of Medicine College, Federal University of Goias, Goiania, Brazil; 4 Biological Sciences and Health Institute, State University of Goias, Goiania, Brazil; 5 Clinics Department of Medicine College, University of São Paulo, Ribeirão Preto, São Paulo, Brazil; Abu Dhabi University, UNITED ARAB EMIRATES

## Abstract

**Introduction:**

Pancreatic adenocarcinoma is an extremely aggressive neoplasm, with many challenges to be overcome in order to achieve a truly effective treatment. It is characterized by a mostly immunosuppressed environment, with dysfunctional immune cells and active immunoinhibitory pathways that favor tumor evasion and progression. Thus, the study and understanding of the tumor microenvironment and the various cells subtypes and their functional capacities are essential to achieve more effective treatments, especially with the use of new immunotherapeutics.

**Methods:**

Seventy cases of pancreatic adenocarcinoma divided into two groups 43 with resectable disease and 27 with unresectable disease were analyzed using immunohistochemical methods regarding the expression of programmed cell death ligand 1 (PD-L1), programmed cell death ligand 2 (PD-L2), and human leukocyte antigen G (HLA-G) molecules as well as the populations of CD4+ and CD8+ T lymphocytes, regulatory T cells (Tregs), and M2 macrophages (MM2). Several statistical tests, including multivariate analyses, were performed to examine how those immune cells and immunoinhibitory molecules impact the evolution and prognosis of pancreatic adenocarcinoma.

**Results:**

CD8+ T lymphocytes and M2 macrophages predominated in the group operated on, and PD-L2 expression predominated in the unresectable group. PD-L2 was associated with T stage, lymph node metastasis, and clinical staging, while in survival analysis, PD-L2 and HLA-G were associated with a shorter survival. In the inoperable cases, Tregs cells, MM2, PD-L1, PD-L2, and HLA-G were positively correlated.

**Conclusions:**

PD-L2 and HLA-G expression correlated with worse survival in the cases studied. Tumor microenvironment was characterized by a tolerant and immunosuppressed pattern, mainly in unresectable lesions, where a broad positive influence was observed between immunoinhibitory cells and immune checkpoint proteins expressed by tumor cells.

## Introduction

Pancreatic adenocarcinoma (PC) is a neoplasm with a very poor prognosis, presenting high morbidity and mortality, with a median five-year survival ranging from 2% to 9% in different countries. It remains one of the most lethal malignant neoplasms, and reports indicate a gradual increase in the number of incident cases and deaths from this disease. This trend of increasing cases of pancreatic cancer is likely to continue as the population ages [1, 2].

Pancreatic neoplasm is characterized by a reduced antigenicity and immunosuppressed microenvironment, with complex interactions between the tumor immune cells and stromal cells in a dense desmoplastic stroma. To overcome immunosurveillance and protect itself, the tumor can use several mechanisms, including the expression of immune checkpoint ligands, secretion of growth factors with immunosuppressive activity, and interference with antigen-presenting cell activity via major histocompatibility complex (MHC) [3, 4].

In addition to the dysfunctional immune system, pancreatic cancer has an overdeveloped dense stroma that functions as a physical barrier, making it difficult for T lymphocytes to access tumor cells. In these circumstances, the stromal barrier also limits the concentration of chemotherapy drugs in these areas, impairing the response to treatment [5, 6].

Although surgery remains the only option with curative potential for treating pancreatic adenocarcinoma, only 10%–15% of patients are eligible for surgical treatment. Early lesions are clinically silent, and by the time of the diagnosis, most cases present at an advanced stage, compromising surgical resection. Others current therapies remain minimally effective. Combined chemotherapy and chemoradiotheray treatments have shown modest improvements in prognosis. More efficient therapeutic modalities for such tumors need to be developed [7].

In recent decades, immunotherapy has become an established therapeutic option for many types of cancer. Unprecedented results of applying immunotherapy, especially using immune checkpoint blockers, have revolutionized cancer treatment. Although lasting responses have been observed in different types of tumors, the results for pancreatic adenocarcinoma have been unsatisfactory, which reflects the tumor’s extreme ability to evade the immune system [8–10].

In the past two decades, several tumoral immune evasion pathways have been discovered, including immune checkpoints. Neoplastic cells express immunoinhibitory molecules (immune checkpoints), such as programmed death ligand-1 (PD-L1), programmed death ligand-2 (PD-L2), and human leukocyte antigen-G (HLA-G) that make them more resistant to attacks by specific tumor T cells. The latter have their functions depressed or blocked in these circumstances [11–13].

In physiological conditions, immune checkpoints are important immunoinhibitory pathways for maintaining self-tolerance and limiting tissue damage that can occur during a habitual immune response [4, 14]. Under these circumstances a balanced response between co-stimulatory and inhibitory pathways occurs. Inhibitory signals, which constitute the various immune checkpoints, strictly control the magnitude and duration of the T cell response to prevent autoimmune injury. However, in the tumor environment, profound and complex transformations occur in which structural, cellular, and biochemical changes that may favor the expression of immune checkpoint by neoplastic cells as an important mechanism of immune evasion and resistance [4].

The most recent immunotherapeutic treatments are specifically aimed at blocking the programmed cell death protein 1 (PD-1) pathway to active anti-tumor immunity. PD-1 is an important inhibitory receptor expressed on T cells following activation. The two ligands for this receptor are programmed cell death ligand 1 (PD-L1) and programmed cell death ligand 2 (PD-L2) [14]. The interaction between them results in the dephosphorylation and attenuation of important molecules in the T cell receptor and CD28 pathways, which inhibits T cell proliferation, decreases activation, lowers cytokine production, alters metabolism, impairs the effector functions of cytotoxic T lymphocytes, and eventually kills activated T cells. Tumor cells, however, use inhibitory pathways to escape immunosurveillance, one of the main ones being the overexpression of PD-L1 and PD-L2 ligands [15, 16].

The intra-tumoral expression of the PD-L1, the main ligand of PD-1, is dynamic and heterogeneous in different types of cancer, including pancreatic cancers, and has been associated with a poor prognosis in most cases [11, 13]. Although PD-L1 may play a predominant role in the PD-1 pathway, PD-L2, the other PD-1 ligand, has been undervalued and far less investigated. PD-L2 expression has been correlated in some studies with clinical response to anti-PD-1 therapy, suggesting a predictive value for this molecule [17]. A meta-analysis study has revealed that PD-L2’s expression may correlate to metastatic potential and predict a reserved prognosis, thus representing a potential target for immunotherapy [12]. However, conflicting results of the blocking of these ligands have also been observed in different types of neoplasms, and its role is still largely unknown in solid tumors. Much more research is needed to understand its full significance [7, 12, 18].

Another common mechanism used by cancer cells to evade the immune system is HLA-G expression, a molecule of the major histocompatibility complex (MHC), with immunosuppressive activity and restricted expression in healthy individuals. In pregnant women, for instance, it plays an important role in fetal immune tolerance. However, in neoplastic processes, that immunotolerant molecule is related to poor prognoses, low survival rates, and advanced tumor stages [19, 20]. Its expression in cancer is heterogeneous and can be used by neoplastic cells to protect themselves from lysis by natural killer cells. The main role of this molecule is the modulation of the immune response, and its blockade may represent an important antitumor strategy [21].

HLA-G binds to various receptors on cells of lymphoid and myeloid lineage, especially to immunoglobulin-like transcripts, where it can function as an immune checkpoint. Over the years, many studies have shown HLA-G’s ectopic expression in various types of cancer [20, 21].

The positive association between the expression of pro-inflammatory transcripts and HLA-G may indicate that HLA-G is a counter-regulatory mechanism that follows the intra-tumoral infiltration of tumor-reactive lymphocytes. The expression of immune checkpoints, such as PD-1, cytotoxic T lymphocyte-associated protein 4 (CTLA4), and indoleamine 2, 3-dioxygenase (IDO), also has a high correlation with pro-inflammatory transcripts. These data indicate that HLA-G can be upregulated in tumor cells to suppress the tumor-induced immune response; thereby modulating the phenotype and function of immune cells. This dynamic can also allow tumor cells to evade immune attack as a greater number of cancer cells express HLA-G in order to protect themselves [22–24].

A very peculiar feature of pancreatic adenocarcinoma is its abundant and hypoxic desmoplastic stroma, which may represent up to 80% of the tumor volume. It functions as a very active niche with mixed stimuli, both stimulatory and inhibitory, creating conditions for reversible changes in various types of tumor-infiltrating immune cells. Complex crosstalk is observed between subtypes of immune cells, tumor cells, and stromal cells with each other, establishing a wide network of intercellular communication and interference in this environment. Cancer associated fibroblasts (CAF) play a very important role in building up this stroma. These cells once activated, produce large amounts of extracellular matrix proteins and stimulate the secretion of cytokines and chemokines that act ultimately, facilitate tumor evasion and dissemination [5, 6, 25].

The immune infiltrate in pancreatic cancer is heterogeneous and, has not been well characterized. Some research has documented a significant immune cell infiltrate, with a predominance of tumor-associated macrophages (TAM), neutrophils, myeloid-derived suppressor cells, and regulatory T cells. These cells play a predominant pro-tumor role that correlates with poor prognosis [26, 27].

Tumor associated macrophages are described as key regulators of the neoplastic environment, and may protect tumor cells from attack by cytotoxic T lymphocytes. They have functional plasticity with phenotypic variation as the tumor progresses, with M1 macrophage characterized by a pro-inflammatory, anti-tumor profile and M2 macrophage, by an anti-inflammatory, protumor profile [28]. The final subtype of these cells is determined by signals from the microenvironment triggered by cytokines, chemokines, vascular endothelial growth factor, and colony-stimulating factors of monocytes and granulocytes [28, 29].

Other important cells in the context of tumor development are regulatory T cells (Tregs), which are known to suppress the antitumor immune response mediated by effector T lymphocytes. Intratumoral Tregs cells are usually increased in the microenvironment of most solid tumors, including pancreatic carcinoma, in which they are most often associated with poor prognoses [30]. Nonetheless, the exact immunomodulatory function of those cells is still poorly understood in these cases. Some mechanisms involved include directly eliminating effector T cells, blocking the activity of antigen-presenting cells, and blocking the production of pro-inflammatory cytokines [31]. Studies have also shown that, in the neoplastic microenvironment, the Treg cells can hyper-regulate HLA-G molecules, which consequently favor the differentiation of Treg cells in a feedback loop [31, 32].

Although cancer is usually characterized by an immunosuppressed milieu, it can also have an effector immune infiltrate represented predominantly by CD8+ cytotoxic T lymphocytes and CD4+ helper T lymphocytes. Such cells are mediators of anti-tumor immunity. Whereas the former is characterized by an ability to directly recognize and eliminate cells expressing tumor-specific antigens, the latter are characterized by an ability to coordinate various immune responses that integrate adaptive and innate effector mechanisms, necessary for balanced and efficient immune activity [26].

In the neoplastic environment, the efficient immune response can be replaced by a nonprotective immune state, with tumor escape from immunological detection and elimination. Thus, the aggressive behavior of the tumor occurs not only due to by the activity of tumor cells, but also by presence of dysfunctional immune cells and immunoinhibitory pathways that favor tumor grow and invasion [14, 26].

In our study, the tumor microenvironment (i.e., immune cells and immune-inhibitory molecules) was analyzed in two groups of patients with pancreatic adenocarcinoma: patients who were operated on (i.e., resected) and patients who were not (i.e., unresectable). The associations between the density and distribution of immune cells, as well as between the expression of immune checkpoint molecules and clinical and pathological characteristics were studied, as were the correlations of those immune inhibitory molecules with patients’ overall survival and their impact on prognosis.

## Materials and methods

A cross-sectional analytical study is presented herein, conducted at the Clinical Hospital of Federal University of Goias (in Portuguese: Hospital das Clínicas da Universidade Federal de Goiás—HC/UFG), Opinion number 2839739, with participation of the Cancer Combat Association in Goias (in Portuguese: Associação de Combate ao Câncer de Goiás—ACCG), Opinion number 2882785. All data were fully anonymized before being accessed. The ethics committees waived the requirement for informed consent. The dates on which the data for research purposes have been accessed correspond to the period from 05/17/2019 to 06/28/2019.

The data supporting this research, along with the ethics committee approval documents, are deposited in the Harvard Dataverse repository (DOI: http://doi.org/10.7910/DVN/UJQ7ZW).

### Case selection

Selection included 70 patients diagnosed with pancreatic ductal adenocarcinoma divided into two groups: 43 cases of resectable tumors, and 27 cases of unresectable tumors. The unresectable cases were collected by a core needle biopsy. All samples were obtained from prancreatic head exclusively. The resectability of the tumor in each patient was determined on imaging studies performed by experienced radiologists.

Corresponding pathologic reports, slides, and paraffin-embedded tissue blocks for all tumors were analyzed. Only patients without any history of neoadjuvant therapy were included in the study.

### Histomorphological analysis

Cases included in the study were reviewed simultaneously by 2 pathologists (MAPCC and MARM), using the updated staging according to the 8th edition of the classification of tumors proposed by the American Joint Committee on Cancer (AJCC) [33].

### Immunohistochemical method

Paraffin blocks from incisional biopsies (i.e., patients not operated on) and the products of the Whipple procedure were used. An immunohistochemical analysis was performed to evaluate the presence of various immune cells, namely CD4-positive T lymphocytes (Novocastra, 1F6), CD8-positive T lymphocytes (Zeta, C8/144B), CD163-positive M2 macrophages (BioCare, 10D6), and Fox P3-positive Tregs (BioCare, 86D). The tumor expression of PD-L1 (BioCare, CAL10), PD-L2 (Sigma-Aldrich, polyclonal), and HLA-G (Exbio, MEM-G/2) molecules was also evaluated.

The slides of the analysis were read by two experienced pathologists using a five-headed 40× optical microscope (Olympus) 0.53 mm in diameter and with a 10× eyepiece. First, using 10× magnification, three fields with the greatest expression were selected. Subsequently, 40× magnification was used to perform the absolute count of cells expressing CD4, CD8, CD163, and FoxP3. For PD-L1, PD-L2, and HLA-G molecules, the total tumor area of the histological section was defined with a 4× objective, and in the same histological area, the percentage of immunostained tumor was evaluated.

Added to the percentual values, PD-L1, PD-L2, and HLA-G were also categorized dichotomously. The cutoffs were chosen following systematic research using the PubMed database. For the categorization of PD-L1, values ≥5% of cells positively stained were considered to be positive (i.e., membrane and cytoplasm marking) [13, 27]; for PD-L2, values >10% of cells positively stained were considered to be positive (i.e., predominantly cytoplasmic marking) [12]; and for HLA-G, values >75% of cells positively stained were considered to be diffusely positive (i.e., predominantly cytoplasmic staining) [34]. Laboratory protocols have been deposited in protocols.io (DOI: https://dx.doi.org/10.17504/protocols.io.n92ld8ymxv5b/v1).

### Statistical analysis

The software R (version 3.6.1), IBM® SPSS® Statistics (version 25), and GraphPad Prism (version 8.0.2) were used for statistical analysis. Added to descriptive analyses, univariate analyses were performed and p < .250 was chosen for further analysis in multivariate models using the stepwise method. The pseudo *R*^2^ statistics and outcomes of the Hosmer–Lemeshow tests were used to verify the fit quality of the statistical models. The prognostic value of the categorized expression of PD-L1, PD-L2, and HLA-G was studied using Kaplan–Meier curves and compared using the logrank test. A significance level of 5% (p < .05) was considered.

## Results

The study included samples from 70 patients, who were 62 years old on average at diagnosis (range 35 years to 79 years). The patients’ clinical and pathological data are shown in Tables 1 and 2, respectively.

**Table 1:** Clinical characteristics of participants.

	n	%
Sex		
Male	41	58.6
Female	29	41.4
Alcohol consumption		
Yes	26	43.3
No	34	56.7
Smoking		
Yes	36	60.0
No	24	40.0
Pancreatic pathology background*		
Diabetes	18	85.7
Acute pancreatitis	1	4.8
Chronic pancreatitis	3	14.3
First degree relative with pancreatic cancer background		
Yes	3	5.4
No	53	94.6

*21 patients had pancreatic pathological background–one of them presented a double background (diabetes and chronic pancreatitis), so the percentage value was calculated based on that number.

**Table 2:** Pathological characteristics of cases.

	n	%
T stage		
T1		
T1a	1	1.5
T1c	10	14.5
T2	22	31.9
T3	9	13.0
T4	27	39.1
N stage		
N0	29	69.0
N1	9	21.5
N2	4	9.5
M stage		
M0	62	89.9
M1	7	10.1
Clinical Stage (AJCC)*		
IA	8	11.7
IB	14	20.6
IIA	5	7.4
IIB	8	11.8
III	26	38.2
IV	7	10.3
Histologic grade		
G1	11	16.7
G2	49	74.2
G3	6	9.1
Perineural invasion		
Yes	18	72.0
No	7	28.0

*AJCC—American Joint Committee on Cancer

**Fig 1:**
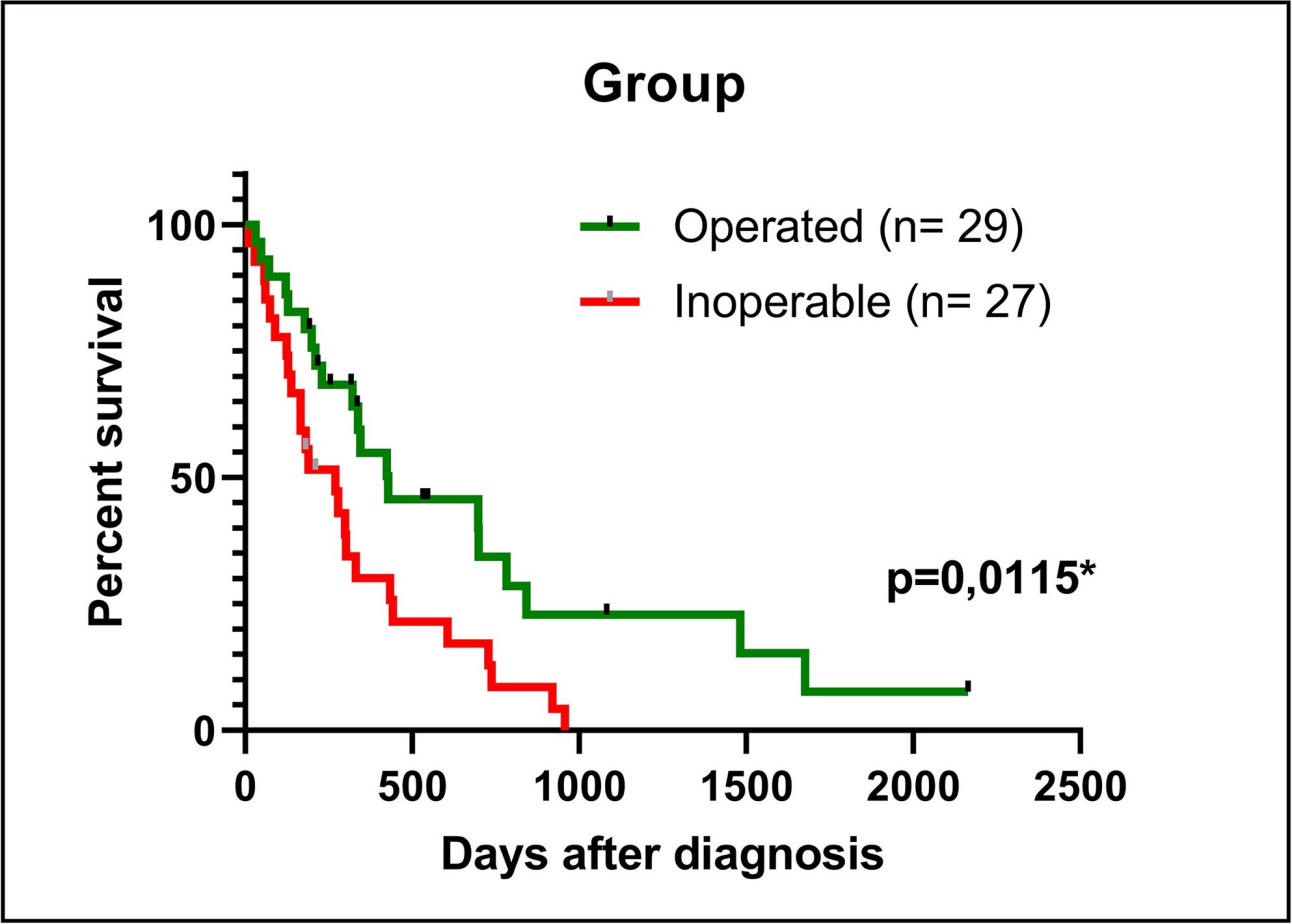
Kaplan-Meier survival curves for operated and inoperable patients.

The average survival of patients operated on was 706 days (median = 425 days), with cumulative survival rates of 55% in Year 1, 22% in Year 3, and 7% in Year 5. It was possible to evaluate the survival of 56 patients, as 14 patients were lost to clinical follow-up. Regarding patients not operated on, the mean survival was 324 days (median = 270 days), with cumulative survival rates of 31% in Year 1, 13% in Year 2, and 0% in Year 3. Statistically significant differences emerged between the groups (*p =* .0115), as shown in Fig 1.

### Immune cells: CD4+ and CD8+ T lymphocytes, FoxP3+ Tregs cells, and CD163+ M2 macrophages

**Fig 2:**
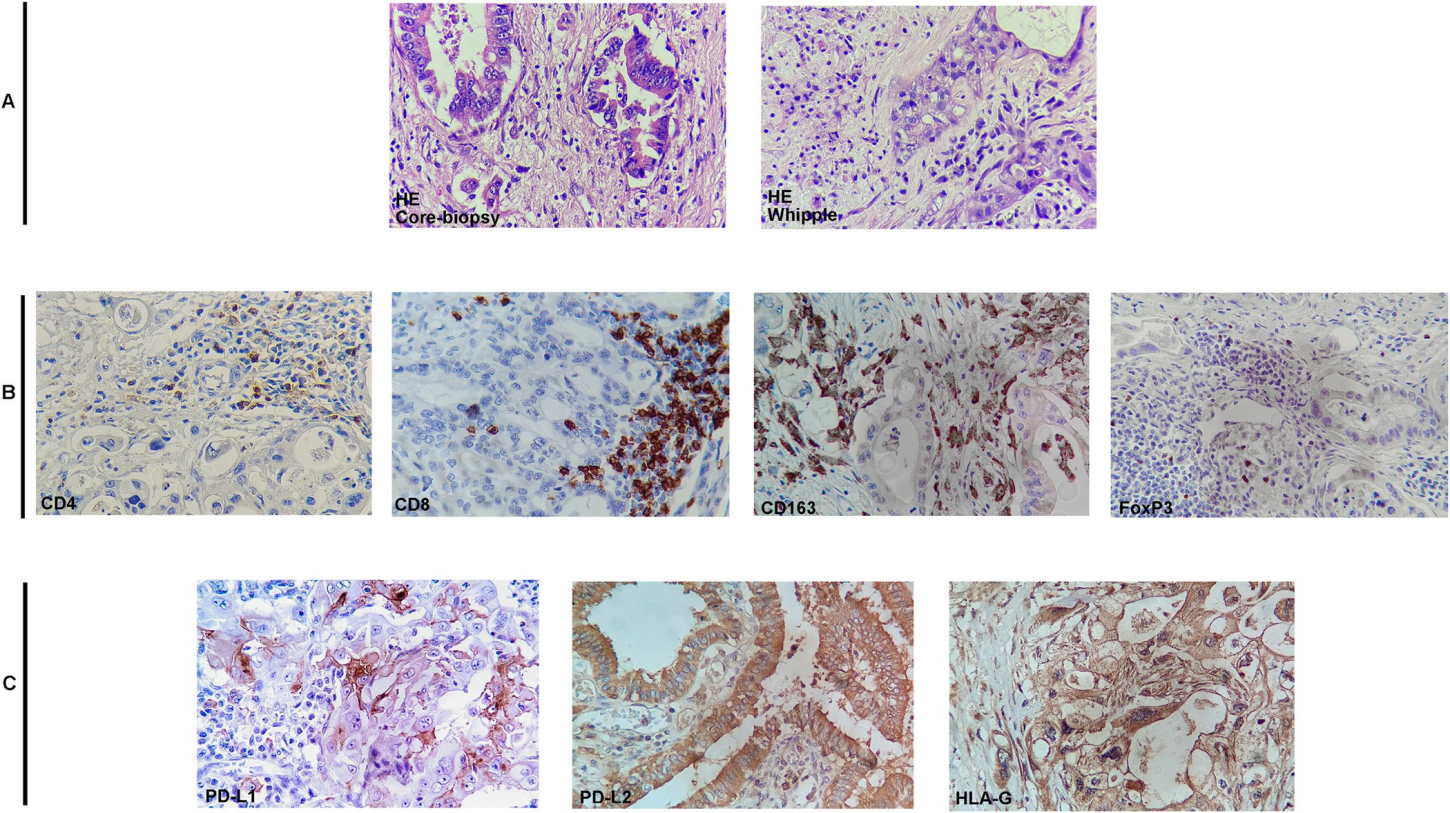
Immune cells and immunoinhibitory ligands expression in pancreatic adenocarcinoma (PDA). **A**, Pancreatic Adenocarcinoma in the infiltrating fronts—HE (400x). **B**, Representative images of immunohistochemical study in PDA lesions: CD4, CD8, CD163 and FoxP3 (400x). **C**, Representative immunohistochemistry (IHC) of immunoinhibitory ligands in PDA (400x).

The distribution of immune cells is presented in Fig 2. There was a statistically significant difference in the number of CD8+ T cells and CD163+ M2 macrophages (*p =* .001 and *p =* .035, respectively) and in relation to tumor T stage (*p =* .011 and *p =* .009 respectively) between resectable and unresectable cases (Table 3). There was also a positive correlation between tumor size and the number of CD163+ M2 macrophages (*p =* .005) and a positive association between the number of FoxP3+ Treg cells and the histological grade of the tumors (*p =* .042). Furthermore, multivariate analysis revealed that patients operated on had 43% more CD8+ T lymphocytes than those not operated on (OR 1.43; 95% CI 1.16–1.76; *p =* .002) and that each centimeter increase in tumor size correlated with an increase in CD163+ macrophages of 9% (OR 1.09; 95% CI 1.02–1.17; *p =* .016). The number of CD4+ T lymphocytes did not show significant association with any of the variables studied.

**Table 3:** Univariate analysis and multivariate evaluation (Quasi-Poisson regression) of clinicopathological features by quantity of intratumor immune cells.

	CD4				CD8				CD163				FOXP3			
	Univariate		Multivariate^7^		Univariate		Multivariate^7^		Univariate		Multivariate^7^		Univariate		Multivariate^7^	
**Categorical variables** ^**8**^	Mean	*p*	OR (95% CI)	*p*	Mean	*p*	OR (95% CI)	*p*	Mean	*p*	OR (95% CI)	*p*	Mean	*p*	OR (95% CI)	*p*
Patient group																
Inoperable (n = 27)	190.6	0.197^1,5^			317.5	**0.001** ^1^	-	-	338.4	**0.035** ^1,5^			51.7	0.522^1^		
Operated (n = 43)	236.9				453.4		1.43 (1.16; 1.76)	**0.002**	412.1				50.2			
T stage																
T1 (n = 11)	225.4	0.450^2^			465.4	**0.011** ^2,6^			336.6	**0.009** ^2,6^			47.3	0.499^2^		
T2 (n = 22)	245.6				461.2				424.2				55.1			
T3 (n = 9)	240.0				398.8				471.7				43.4			
T4 (n = 27)	190.6				317.5				338.4				51.7			
N stage																
N0 (n = 29)	258.6	0.088^2,5^			444.4	0.873^2^			410.8	0.706^2^			50.1	0.948^2^		
N1 (n = 9)	228.3				468.2				431.4				44.9			
N2 (n = 4)	121.8				438.5				370.8				66.0			
N metastasis																
Negative (N0) (n = 29)	258.6	0.161^1^	-	-	444.4	0.624^1^			410.8	0.754^1^			50.1	0.924^1^		
Positive (N1-N2) (n = 13)	195.5		1.00 (0.52; 1.10)	0.154	459.1				412.8				51.4			
M stage																
M0 (n = 62)	224.2	0.538^1^			397.5	0.858^1^			387.8	0.684^1^			52.1	0.758^1^		
M1 (n = 7)	181.1				403.7				367.6				40.6			
AJCC stage group																
I (n = 22)	250.9	0.288^2^			443.3	0.117^2,6^			402.8	0.106^2,6^			47.9	0.978^2^		
II (n = 13)	255.3				431.4				451.8				47.1			
III (n = 26)	189.0				332.5				341.0				58.6			
IV (n = 7)	181.1				403.7				367.6				40.6			
Histologic grade																
G1 (n = 11)	194.8	0.453^2^			345.0	0.642^2^			427.1	0.319^2^			26.3	**0.042** ^2,6^		
G2 (n = 49)	234.7				418.1				391.1				59.3			
G3 (n = 6)	174.8				407.7				316.7				32.3			
Perineural invasion																
Positive (n = 18)	251.3	0.976^1^			499.1	0.739^1^			425.9	0.762^1^			63.6	0.303^1^		
Negative (n = 7)	260.6				455.9				394.0				51.0			
**Numerical variables**	ρ^3^	*p*	OR (95% CI)	*p*	ρ^3^	*p*	OR (95% CI)	*p*	ρ^3^	*p*	OR (95% CI)	*p*	ρ^3^	*p*	OR (95% CI)	*p*
Tumoral size (centimeters)	0.10	0.518			-0.13	0.408			0.42	**0.005**	1.09 (1.02; 1.17)	**0.016**	-0.02	0.892		
Survival days (inoperable) ^4^	0.10	0.642			-0.20	0.332			-0.21	0.305			-0.16	0.443		
Survival days (operated) ^4^	0.15	0.538			-0.07	0.755			-0.13	0.582			-0.39	0.091	1.00 (1.00; 1.00)	0.177

^1^Mann-Whitney test.

^2^Kruskall-Wallis test.

^3^Spearman’s rank correlation coefficient.

^4^Excluded those patients who died by surgical complication and those who was censored.

^5^Variable not included in the multivariate analysis due the construction of the model (variability and/or multicollinearity problems).

^6^Variable included at the initial multivariate model, but who had not statistical significance to proceed to the final model.

^7^Only the final model of multivariate analysis was presented.

^8^Some groups within categorical variables have n lower than the total of 70 due to the lack of information in the histopathological reports.

### Percentage evaluation of PD-L1, PD-L2, and HLA-G molecules

Patients not operated on tended to have higher PD-L2 values than the patients operated on (*p =* .017). PD-L2’s expression was also positively correlated with T stage (*p =* .006), N stage (*p =* .038), and clinical stage (*p =* .013). PD-L1’s expression was also correlated with a worse survival rate among patients operated on (*p =* .037), as shown in Table 4. No association surfaced between HLA-G and the pathological characteristics studied.

**Table 4:** Univariate analysis and multivariate evaluation (simple linear regression) of clinicopathological features by intratumoral expression of immune checkpoints molecules (percentage).

	PD-L1				PD-L2				HLA-G			
	Univariate		Multivariate ^7^		Univariate		Multivariate ^7^		Univariate		Multivariate ^7^	
**Categorical variables** ^**8**^	Mean	*p*	β (95% IC)	*p*	Mean	*p*	β (95% IC)	*p*	Mean	*p*	β (95% IC)	*p*
Patient group												
Inoperable	9.2	0.834^1^			61.7	**0.017** ^1,5^			61.2	0.899^1^		
Operated	6.4				46.2				63.8			
T stage												
T1	2.6	0.611^2^			31.4	**0.006** ^2,6^			63.6	0.991^2^		
T2	9.4				57.3				63.7			
T3	4.6				34.4				61.4			
T4	9.2				61.7				61.2			
N stage												
N0	4.5	0.072^2,6^			41.2	**0.038** ^2,5^			60.0	0.246^2,6^		
N1	15.4				46.1				65.6			
N2	1.8				76.3				81.3			
N metastasis												
Negative (N0)	4.5	0.051^1,5^			41.2	0.112^1^	-	-	60.0	0.312^1^		
Positive (N1-N2)	11.2				55.4		14.18 (-3.21; 31.57)	0.118	70.4			
M stage												
M0	6.8	0.083^1,6^			51.1	0.849^1^			61.7	0.698^1^		
M1	14.6				57.9				70.1			
AJCC stage group												
I	4.9	0.327^2^			42.1	**0.013** ^2,6^			62.1	0.946^2^		
II	10.6				40.4				61.2			
III	6.7				63.5				60.6			
IV	14.6				57.9				70.1			
Histologic grade												
G1	8.6	0.687^2^			37.7	0.241^2,6^			63.2	0.929^2^		
G2	7.8				55.1				65.0			
G3	5.0				56.7				52.8			
Perineural invasion												
Positive	7.9	0.756^1^			45.6	0.329^1^			63.8	0.927^1^		
Negative	8.7				52.9				62.9			
**Numerical variables**	ρ^3^	*p*	β (95% IC)	*p*	ρ^3^	*P*	β (95% IC)	*p*	ρ^3^	*p*	β (95% IC)	*p*
Tumoral size (centimeters)	-0.02	0.907			0.09	0.571			-0.06	0.714		
Survival days (inoperable) ^4^	0.16	0.454			-0.14	0.501			-0.29	0.163^5^		
Survival days (operated) ^4^	-0.38	0.099	-0.01 (-0.018; -0.001)	**0.037**	-0.14	0.559			-0.38	0.097	-0.01 (-0.03; 0.01)	0.212

^1^Mann-Whitney test.

^2^Kruskall-Wallis test.

^3^Spearman’s rank correlation coefficient.

^4^Excluded those patients who died by surgical complication (30 days) and those who was censored.

^5^Variable not included in the multivariate analysis due the construction of the model (variability and/or multicollinearity).

^6^Variable included at the initial multivariate model, but who had not statistical significance to proceed to the final model.

^7^Only the final model of multivariate analysis was presented.

^8^Absolute values for the categorical variables were presented in Table 3.

### Dichotomous evaluation of PD-L1, PD-L2, and HLA-G molecules

As shown in Table 5, the dichotomous analysis of the results of PD-L1, PD-L2, and HLA-G revealed that of the 70 patients studied, 21 (30.0%) were positive for PD-L1, 59 (84.3%) for PD-L2, and 32 (45.7%) for HLA-G. The positivity and expression patterns of those molecules are depicted in Fig 2. There was also a univariate association between PD-L1’s expression and N-stage (*p =* .030) between PD-L2 and T-staging (*p =* .035) and between PD-L2 and survival in patients not operated on (*p =* .031).

**Table 5:** Univariate analysis and multivariate evaluation (Quasi-Poisson regression) of clinicopathological features by dichotomous intratumoral expression of immune checkpoint molecules.

	PD-L1							PD-L2							HLA-G						
	Univariate					Multivariate ^7^		Univariate					Multivariate ^7^		Univariate					Multivariate ^7^	
	Negative		Positive		*p*	OR (95% CI)	*p*	Negative		Positive		*p*	OR (95% IC)	*p*	Negative		Positive		*p*	OR (95% IC)	*p*
**Categorical variables**	n	%	n	%				n	%	n	%				n	%	n	%			
Patient group																					
Inoperable	18	66.7	9	33.3	0.994^1^			3	11.1	24	88.9	0.511^2^			15	55.6	12	44.4	1.000^1^		
Operated	30	69.8	13	30.2				8	18.6	35	81.4				23	53.5	20	46.5			
T stage																					
T1	10	90.9	1	9.1	0.322^2^			4	36.3	7	63.7	**0.035** ^2,5^			6	54.5	5	45.5	0.867^2^		
T2	13	59.1	9	40.9				1	4.5	21	95.5				11	50	11	50			
T3	6	66.7	3	33.3				3	33.3	6	66.7				6	66.7	3	33.3			
T4	18	66.7	9	33.3				3	11.1	24	88.9				15	55.6	12	44.4			
N stage																					
N0	23	79.3	6	20.7	**0.030** ^2,6^			6	20.7	23	79.3	1.000^2^			17	58.6	12	41.4	0.546^2^		
N1	3	33.3	6	66.7				2	22.2	7	77.8				5	55.6	4	44.4			
N2	3	75	1	25				0	0	4	100				1	25	3	75			
N metastasis																					
Negative (N0)	23	79.3	6	20.7	0.068^2,5^			6	20.7	23	79.3	1.000^2^			17	58.6	12	41.4	0.516^2^		
Positive (N1-N2)	6	46.2	7	53.8				2	15.4	11	84.6				6	46.2	7	53.8			
M stage																					
M0	44	71.0	18	29.0	0.198^2,6^			11	17.7	51	82.3	0.587^2^			34	54.8	28	45.2	1.000^2^		
M1	3	42.9	4	57.1				0	0	7	100				4	57.1	3	42.9			
AJCC stage group																					
I	17	77.3	5	22.7	0.230^2,5^			4	18.2	18	81.8	0.326^2^			12	54.5	10	45.5	0.967^2^		
II	7	53.9	6	46.1				4	30.8	9	69.2				8	61.5	5	38.5			
III	19	73.1	7	26.9				3	11.5	23	88.5				14	53.8	12	46.2			
IV	3	42.9	4	57.1				0	0	7	100				4	57.1	3	42.9			
Histologic grade																					
G1	8	72.7	3	27.3	1.000^2^			4	36.4	7	63.6	0.072^2,6^			7	63.6	4	36.4	0.776^2^		
G2	33	67.3	16	32.7				5	10.2	44	89.8				25	51.0	24	49.0			
G3	4	66.7	2	33.3				1	16.7	5	83.3				3	50	3	50			
Perineural invasion																					
Positive	11	61.1	7	38.9	1.000^2^			2	11.1	16	88.9	0.548^2^			9	50	9	50	0.407^2^		
Negative	4	57.1	3	42.9				2	28.6	5	71.4				5	71.4	2	28.6			
**Numerical variables**	n	Mean	n	Mean	*p*	OR (95% IC)	*p*	n	Mean	n	Mean	*p*	OR (95% IC)	*p*	n	Mean	n	Mean	*p*	OR (95% IC)	*p*
Tumoral size (centimeters)	29	3.14	13	3.51	0.318^3^			8	3.24	34	3.26	0.664^3^			23	3.37	19	3.12	0.694^3^		
Survival days (inoperable) ^4^	17	273.65	8	383.63	0.294^3^			2	848.0	23	261.96	**0.031** ^3^	0.99 (0.98; 1.00)	0.099	14	366.64	11	235.27	0.352^3^		
Survival days (operated) ^4^	15	554.87	5	186.2	0.081^3^	1.00 (0.99; 1.00)	0.152	2	740.0	18	431.89	0.186^3,5^			8	605.25	12	367.67	0.203^3^	1.00 (1.00; 1.00)	0.265

^1^Chi-squared test.

^2^Fisher’s exact test.

^3^Mann-Whitney test.

^4^Excluded those patients who died by surgical complication (30 days) and those who was censored.

^5^Variable not included in the multivariate analysis due the construction of the model (variability and/or multicollinearity).

^6^Variable included at the initial multivariate model, but who had not statistical significance to proceed to the final model.

^7^Only the final model of multivariate analysis was presented.

### Survival curves and PD-L1, PD-L2, and HLA-G molecules

**Fig 3:**
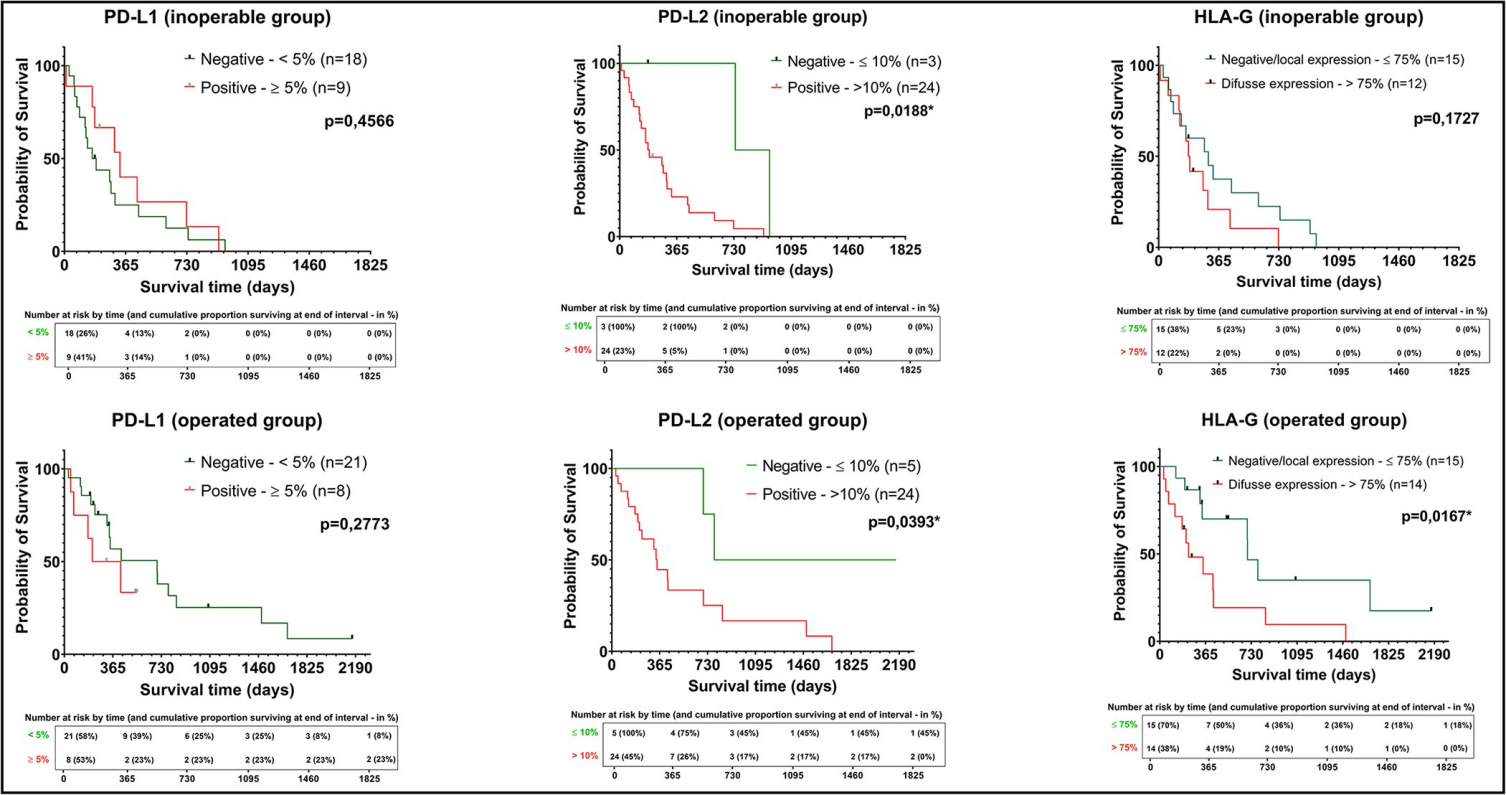
Kaplan-Meier curves and five-year risk tables, in addition to cumulative proportion surviving percentage, regarding dichotomous evaluation of immune checkpoints (PD-L1, PD-L2, and HLA-G) in both inoperable and operated groups.

A statistically significant difference arose between PD-L2’s expression and survival in both groups. A worse outcome was observed in patients who were positive for PD-L2 than in ones who were negative for it (Fig 3). HLA-G’s expression was also significantly different within the group of patients operated on. A worse outcome was observed in patients who were positive for HLA-G than in those who tested negatively (Fig 3). No significant correlation arose between PD-L1’s expression and the survival curve in either group.

### Correlation between the density of immune cells and immunosuppressive molecules in patients operated on and not operated on

**Fig 4:**
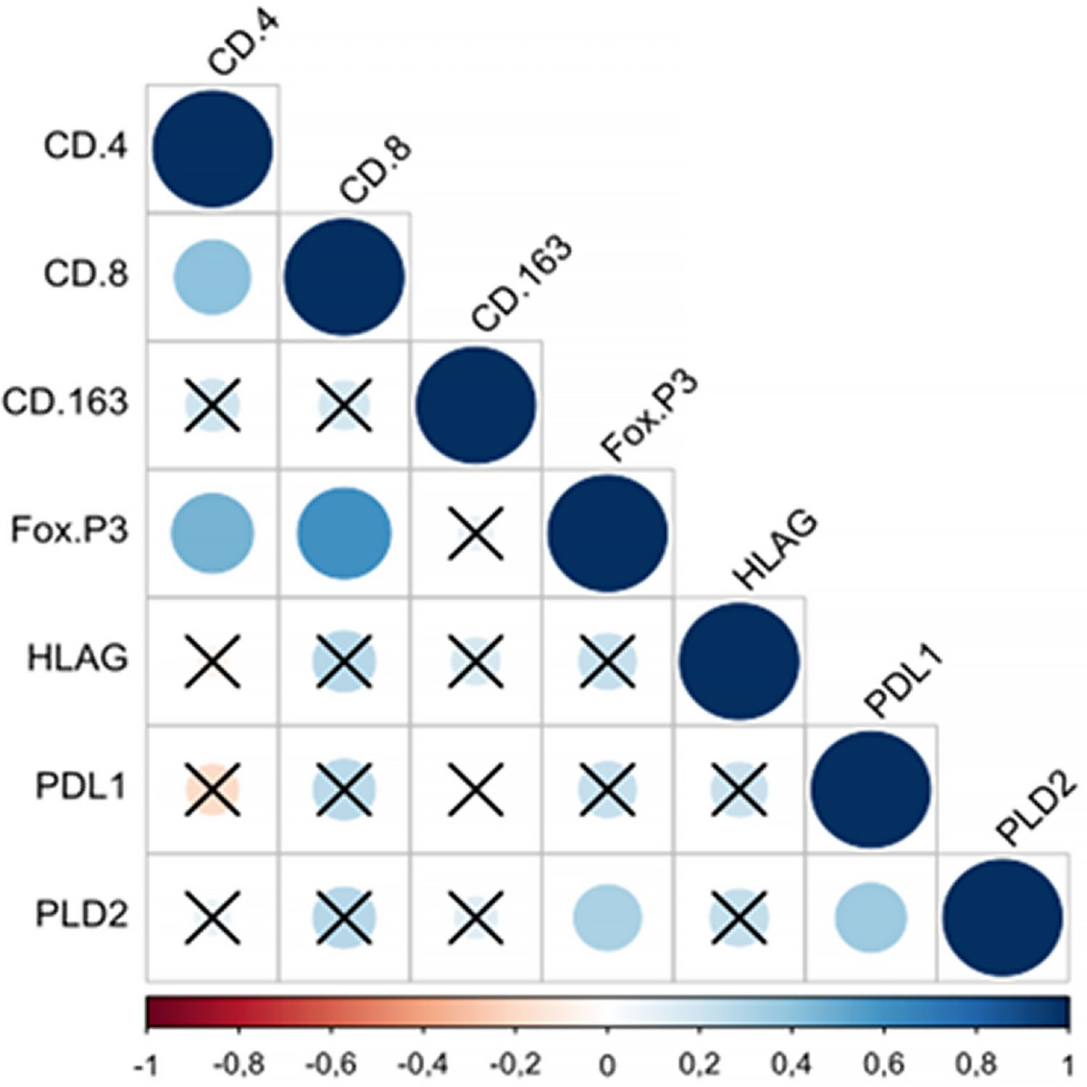
Spearman’s rank correlation coefficient in operated patients. Significant correlations can be observed in spheres that do not have an X. The larger and bluer the sphere, the greater the positive correlation between the variables. The negative correlation is represented by the red sphere. The larger the sphere and the more intense its red color, the greater the negative correlation between the variables. In operated patients, it can be observed that the most significant positive correlation occurred between the CD8 and FoxP3 variables. Therefore, when CD8 values increase, FoxP3 values also tend to increase. In this group of patients, no significant negative correlation was observed.

In the patients operated on, as shown in Fig 4, significant positive correlations arose between CD4 and CD8 (*p* = .007), CD4 and FoxP3 (*p =* .002), CD8 and FoxP3 (*p* < .01), FoxP3 and PD-L2 (*p =* .037), and PD-L1 and PD-L2 (*p =* .021).

**Fig 5:**
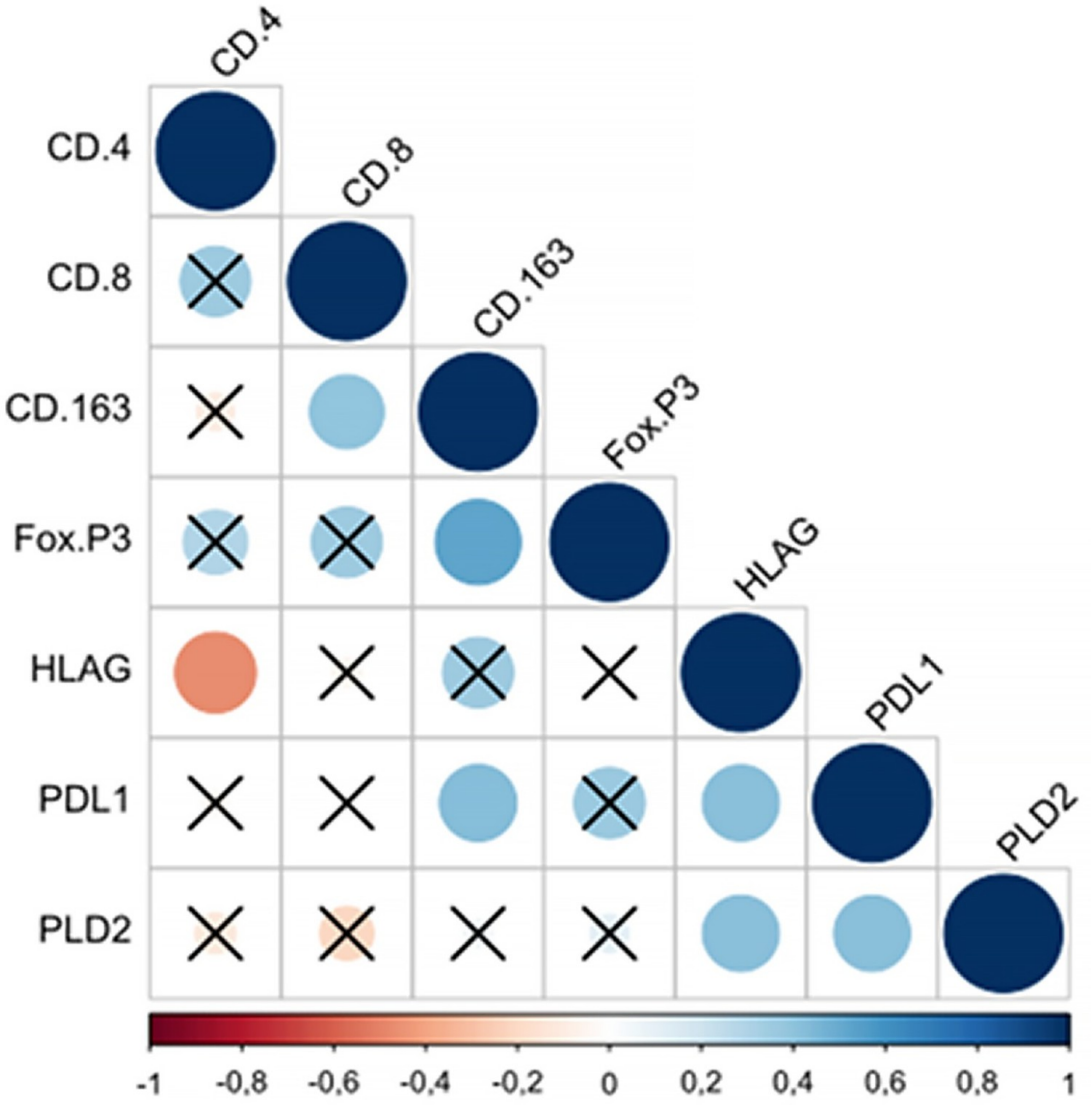
Spearman’s rank correlation coefficient in inoperable patients. Significant correlations can be observed in spheres that do not have an X. The larger and bluer the sphere, the greater the positive correlation between the variables. The negative correlation is represented by the red sphere. The larger the sphere and the more intense its red color, the greater the negative correlation between the variables. In inoperable patients, it can be observed that the most significant positive correlation occurred between the variables CD163 and FoxP3. Therefore, when CD163 values increase, FoxP3 values also tend to increase and vice versa. One can also notice a significant negative correlation between HLA-G and CD4, meaning that an increase in HLA-G expression tends to correlate with a decrease in the number of CD4 positive cells and vice versa.

Among patients not operated on (Fig 5), significant positive correlations emerged between CD8 and CD163 (*p =* .042), CD163 and Fox P3 (*p =* .005), CD163 and PD-L1 (*p =* .027), HLA-G and PD-L1 (*p =* .030), HLA-G and PD-L2 (*p =* .031), and PD-L1 and PD-L2 (*p =* .032). A negative correlation was also observed between CD4 and HLA-G (*p =* .014).

## Discussion

In recent decades, immunotherapy has become an established therapeutic option for coping with cancer, and unprecedented results with this type of therapy have revolutionized cancer treatment. Different strategies to stimulate and potentiate the immune response have been tested and include vaccines, immune checkpoint inhibitors, and co-stimulatory pathway agonists [9, 35, 36].

While the treatment of different malignancies has shown important progress with advances in immunotherapy, pancreatic cancer remains poorly responsive to available drug therapies. This reflects the complex tumor microenvironment in this neoplasm and the need for further research [37].

### Immune cell density and its association with clinical-pathological characteristics

Although described as a tumor with low immunogenicity, pancreatic cancer can exhibit an effector immune infiltrate with numerous tumor infiltrating T cells. However, this is dysfunctional, and unable to mount a complete antitumor immune response, blocked by the secretion of immunosuppressive cytokines [38, 39].

In our study, a heterogeneous population of immune cells was observed with a predominance of CD8+ T cells and M2 macrophages in the group of resected patients. These findings are in agreement with other studies showing that even with a suppressive and regulatory immune signature, pancreatic cancer also contains an effector immune infiltrate that fails to act by deactivating the response of cytotoxic T cells or inhibiting their function. In this way, an inert lymphoid infiltrate develops with little or no ability to react [9, 26].

CD8+ T cells were also significantly more numerous among patients operated on with lesser T staging. These findings corroborate past results showing that a greater number of effector T cells correlates with better prognosis and that those cells tend to decrease in number as the neoplasm progresses [6, 40]. With tumor growth, those lymphocytes become unable to interact directly with cancer cells, begin to present a more heterogeneous distribution, and become displaced, mostly to the periphery of the lesion as if excluded from the environment, thereby allowing neoplastic cells to move more freely. Those T cells tend to be more distantly located from tumor cells, and sometimes form lymphoid structures adjacent to the infiltrative margins of the tumor. Many of these cells become trapped in the dense fibrous stromal, with their effector functions progressively blocked [39–42].

Among the different tumor-infiltrating immune cells are myeloid-derived suppressor cells (MDSC). These cells are implicated in immune suppression in cancer and other chronic pathological conditions. They are morphologically and phenotypically similar to neutrophils and monocytes/marcrophages, which makes it difficult to identify these immune cells exclusively by morphology and phenotype. These cells can be differentiated by biochemical and genomic profiles [43].

Another markedly active immune cell in the tumor environment is represented by M2 tumor-associated macrophages. This has been described as a key element of the neoplastic environment, one that has been correlated with tumor growth, invasion, and metastasis in several studies [26, 44].

The population of M2 macrophages, which represented a large part of the leukocytes in our study, predominated in patients who were operated on. These findings suggest that an immunosuppressive state begins to develop early in tumor evolution, with the MM2 being critical for mediating tumor immune escape, thus protecting neoplastic cells from immune pressure. Thus, these cells, despite being able to escape immunosurveillance, keep their antigenicity preserved, and consequently, remain vulnerable to the action of effector T cells [9, 28].

Our research showed a positive correlation between the number of M2 macrophages and the T stage and tumor size. It has been described that as the tumor grows and invades, pro-inflammatory M1 macrophages switch to the M2 phenotype as a result of a Th2-type immune response. There is also a feedback loop between tumor cells and M2 macrophages in an interactive and dynamic process. Neoplastic cells induce M2 macrophages, which consequently promote tumor growth and protect neoplastic cells, such that they are not targeted by cytotoxic T cells or natural killer cells [29, 45, 46]. However, these macrophage cells with their great functional plasticity, have pro-inflammatory potential that can be activated in response to various stimuli, in the neoplastic environment, including production of cytokines, chemokines, and growth factors. All these interactions can lead to conflicting findings regarding its role [28, 29, 47]. Ultimately, a predominant pro-tumor or pro-inflammatory profile closely relates to the type of cytokines and chemokines prevalent in the tumor environment [26].

In the present study, it was expected to find more M2 macrophages in patients with unresectable tumors than those who underwent surgery, since cancer progression is associated with increased immunosuppression and infiltration by numerous myeloid cells [28, 48]. Although the population of M2 macrophages was the largest among all immune cells quantified in the patients with unresectable disease, a comparison of the density of cells between the groups revealed a significantly greater number of M2 macrophages in the group of patients operated on. These findings may be related to the composition of the tumor in inoperable cases, which changes as the neoplasm evolves. In the most advanced cases, there is an abundant stromal component, with a dense network of collagen matrix. Initially, the characteristic desmoplastic stroma represents only 5% of the tumor mass. As the tumor develops, this stromal component may account for up to 90% of tumor volume [49].

In relation to cancer, other extensively investigated immune cells are Treg cells. These cells have been described as a prominent feature in the microenvironment in pancreatic cancer, most often located in the juxtatumoral stroma. The presence of these cells has been correlated with lymph node metastasis and less tumor differentiation and, consequently, with unfavorable prognoses [50]. Although the immunomodulatory function of Treg cells in such neoplasm remains poorly understood, their presence in the neoplastic stroma has been associated with a suppressed T cell-mediated immune response and with impaired immunosurveillance [37]. Increased Treg cells in the tumor have also been correlated with a blockade in the recruitment of CD8+ T cells and inhibition of immunogenic function of antigen-presenting cells [31, 51]. However, conflicting findings between Treg cell density and prognoses have also been described. In a study by Dr. Zhang and colleagues 2020, regulatory T- cell depletion was shown to accelerate the progression of pancreatic cancer [52].

In the present study, an association was observed between Treg cells and histological grade, with more of those cells in grade 2 tumors (i.e., moderately differentiated) than in grade 1 tumors (i.e., well differentiated), which is consistent with the progression of immunosuppressive state during neoplastic evolution. However, that trend was not observed when comparing grade 2 tumors with grade 3 tumors (i.e., poorly differentiated tumors). Previous research also obtained similar conclusions and it was considered that the variability of the cellular phenotype and the biochemical conditions of the microenvironment in tumors of different histological grades, could justify this finding [50].

Other tumor infiltrating lymphocytes of interest were the CD4+ helper T cells. The role of these cells in pancreatic cancer immunity is poorly understood. It is known that the presence of that type of T lymphocyte in the tumor microenvironment may relate to a favorable prognosis, especially if associated with the concomitant presence of CD8+ T cells. However, it often has its proliferation and migration inhibited within the tumor [26, 39]. In this environment, these cells can differentiate into Th1, Th2, Th17, and Tregs depending on the type of predominant cytokines. Potent immunosuppressive cytokines such as interleukin-10 (IL-10) and transforming growth factor-beta (TGF-beta) induce the Th2 phenotype, with the establishment of an immunotolerant pattern within the tumor. The few studies showing the response of those cells to pancreatic adenocarcinoma suggest that the immunity of CD4+ helper T cells in such neoplasm is directed toward a type Th2 suppressive immune response [30, 53, 54]. In our study, no significant association surfaced between the expression of CD4+ T cells and clinical or pathological characteristics.

### Tumor expression of PD-L1, PD-L2, and HLA-G and its association with clinical-pathological features

In our study, both PD-1 ligands (i.e., PD-L1 and PD-L2) were expressed by tumor cells from both groups evaluated, with markedly greater positivity for PD-L2, which suggests the functional relevance to this immune checkpoint molecule [55]. PD-L1 and PD-L2 dominated among patients not operated on, whereas only PD-L2 showed a statistically significant difference in relation to patients operated on. Although PD-L2’s expression is more restricted than PD-L1’s, PD-L2 can bind to its PD-1 receptor with higher affinity than PD-L1, which would make it an interesting target for effector T cells [17, 56].

When all patients were evaluated regarding the positivity rate of PD-L2, the rate was higher than rates described in a few other studies evaluating the same molecule in pancreatic adenocarcinomas [17, 55]. Those different results may be linked to intrinsic aspects of the tumor environment, the time of evolution of the neoplasm, or different evaluation criteria [57].

Research has also shown that PD-L2’s expression is not as restricted as initially thought and that, in pancreatic adenocarcinomas PD-L2 can be hyper-regulated in response to immunosuppressive cytokines released by a Th2-type response, contributing to the deactivation of the anti-tumor immune response [12, 18, 56]. Studies have also suggested that PD-L2 plays a role in tumor immunity by blocking T lymphocytes both in the induction phase and in the effector phase of the anti-tumor immune response. Its expression, regardless of PD-L1, can also be predictive of the response to therapy targeting the PD-1 axis [16, 17].

Regarding clinical and pathological variables, a significant association was observed between PD-L2’s expression with tumor size, lymph node involvement, and clinical staging, along with a clear negative correlation with the mean survival of patients not operated on. That influence was also observed when PD-L2 was analyzed in relation to the survival curve in both groups evaluated. Previous studies have also shown that high levels of intra-tumoral PD-L2 were related to poor survival in pancreatic cancer and other malignancies [12, 16, 58, 59].

These findings suggest that PD-L1 or PD-L2 expression by the tumor cells would carry a worse prognosis and may be useful if PD-1 immune checkpoint blocking therapy were available [56, 60].

In the PD-1 pathway, PD-L1 has been the main ligand investigated in relation to its prognostic role and as a predictive biomarker of response to immunotherapies with immune checkpoint inhibitors. The presence of this molecule has also been associated with poor prognosis for several types of tumors, including pancreatic tumors [61–63]; however, in some studies, its expression in untreated pancreatic carcinoma has been described as weak and infrequent [13, 27]. In line with those findings, we observed that PD-L1 was not hyper-regulated in relation to PD-L2 in either group of patients, but showed focal expression and heterogeneous distribution among neoplastic cells. These results may be related to the irregular distribution of cytotoxic T cells within the neoplastic stroma. In this environment, lymphoid cells are excluded from the areas closest to tumor cell nests. Thus, there is no activation of tumor infiltrating T lymphocytes and, consequently, there is no secretion of interferon gamma, which is essential for the adaptive expression of PD-L1 by tumor cells [39, 60].

The increased expression of PD-L1 in our study was associated with a worse survival rate in the group of patients operated on. In the dichotomized expression (positive or negative) of this molecule, there was a correlation of positive cases with lymph node metastasis, as similarly described by other authors [64–66].

These findings reinforce pancreatic cancer’s extreme ability to evade effector immune cells and, in turn, reflect the complexity of the tumor microenvironment dominated by immunosuppressive cells, immunotolerant cells, and abundant desmoplastic stroma containing various stress factors that alter the phenotype of tumor cells, promoting tumor escape and spread [11, 60, 67]. Between the two groups studied, PD-L1’s expression was greater in unresectable cases; however, the difference was not statistically significant, limiting the evaluation of the molecule in this context.

In our study, despite no significant difference in the expression of HLA-G between the two groups evaluated, a significant correlation between the expression of this molecule and survival was observed. As with PD-L2, HLA-G’s expression correlated with significant impairment in survival (Fig 3). Similar results have been observed in other studies evaluating that molecule’s role in pancreatic cancer [20, 34, 68]. Different from expression of PD-1 pathway ligands, HLA-G’s expression does not depend on the activation of T cells in the context of the adaptive immune response and can block the immune response by inhibiting all immune effectors, from the activation of antigen-presenting cells to the cytolytic function of cytotoxic T lymphocytes and natural killer cells [21, 69, 70].

### Correlations between all variables

As pancreatic cancer develops, the tumor environment becomes increasingly complex and hostile, which significantly disrupts the body’s homeostasis. The rate of tumor growth progressively increases under the influence of the continuous interaction between cancer cells and the host’s immune system. The tumor progressively transforms the environment, leading to important structural and functional alterations [49]. In the tumor niche, important immune cells interact with each other as well as with tumor and stromal cells by different pathways in a rather complex crosstalk. The interchange between those cells establishes the immune status of the tumor microenvironment resulting from the balance of immunostimulatory and immunosuppressive signals, which directly reflects on the patient’s prognosis [6, 26].

In the analysis of the influence or correlation between all variables studied, considering the three immunoinhibitory molecules (i.e., PDL-1, PDL-2, and HLA-G) and the four immune cells (i.e., CD4+ and CD8+ T lymphocytes, Tregs cells, and M2 macrophages), a heterogeneous pattern of interactions was noted, sometimes with discrepancies within and between the two groups of patients evaluated. (Figs 4 and 5).

In the group of patients operated on, a positive reciprocal influence emerged between CD8+ cytotoxic T lymphocytes and CD4+ helper T lymphocytes, one showing that those cells initially work together to maintain active immunosurveillance. At that stage, the immune system demonstrates balanced and efficient functions. That observation is reinforced by past results showing that both CD8+ and CD4+ T cells in pancreatic adenocarcinoma correlate better with the prognosis than when only one of them is detected in the environment [13, 26, 54].

In our research, a significant inverse correlation was also noted between HLA-G and CD4+ helper T cells, one that was highly expressive among patients not operated on. This finding could suggest the influence of HLA-G on the differentiation of subtypes of immune cells, which determines the tolerance for different mechanisms, including the inhibition of helper T cells and the induction of their differentiation into Treg cells in the neoplastic environment [21, 34].

Moreover, a broad positive correlation between immune cells and immunosuppressive molecules was noted in the group of patients not operated on, as shown in Fig 5. PD-L1, PD-L2, and HLA-G showed strong positive and reciprocal influences in that group. These findings could reflect an integrated network of well-established immunosuppressive signals within a complex, active and immunotolerant tumor environment, in patients with advanced disease [7].

In this research, PD-L1 showed a significant positive correlation with M2 macrophages in the inoperable cases. These immune cells interact by various mechanisms, some of which are represented by the secretion of immunosuppressive cytokines and chemokines, and others, by the expression of inhibitory ligands such as PD-L1 on the surface of these macrophages, promoting T cell apoptosis [13, 29].

A strong correlation also emerged between M2 macrophages and Treg cells. These macrophages recruit and favor the local maintenance of Treg cells in addition to inducing the change from CD4+ T cells to Th2 cells and Treg cells that reinforce the immune suppression of the microenvironment [9, 29]. Th2 cells in turn, induce M2 macrophages by the secretion of cytokines such as TGF-beta and IL-10, establishing a feedback mechanism [28, 29].

It can be clearly observed that with tumor progression, an immunosuppressive pattern becomes dominant in the neoplastic microenvironment, due to installation and activation of crucial pathways to tumor growth and evasion. Such conditioning of the environment involves the expression of ligands of immune checkpoint pathways, secretion of pro-tumor cytokines, a varied transit of dysfunctional immune cells, and the activation of mesenchymal stromal cells. The latter are represented by cancer-associated fibroblasts that contribute significantly to the formation of the dense and abundant tumor stroma, characteristic of this neoplasm. These fibroblasts are also versatile and interact with immune cells and tumor cells, contributing to local immunosuppression [26, 39, 49].

In general, the correlations of those variables evaluated, especially in more advanced stages of neoplastic development, may suggest an integrated network of cooperation among all those factors, one aiming to protect the tumor and promote its progression with a complete blockade and deactivation of the anti-tumor immune response. Considering the heterogeneity of the tumor microenvironment, analyzing the expression of these immunoinhibitory cells and molecules with evaluation of the whole set, will significantly increase the predictive value of the study [26, 71].

## Conclusions

Although our study had limitations regarding resources, a small sample, and the limited clinical reports available, it did show some interesting data.

A more aggressive evolution was observed in the group with unresectable disease, with no patients alive in the third year after diagnosis. A low histological grade of the tumor was observed in almost all operated cases, while approximately 84% of unresectable cases had a high histological grade. The expression of PD-L2 and HLA-G correlated with a decrease in overall survival, and resulted in an unfavorable prognosis in these cases.

Higher PD-L2 expression and higher number of M2 macrophages were associated with higher tumor staging, suggesting that an immunosuppressed environment favors tumor growth. The neoplastic environment appears to become progressively more immunosuppressed and immunotolerant as the tumor advances, with a strong positive influence among the immunoinhibitory molecules PD-L1, PD-L2 and HLA-G in the non-operated on group.

In addition, the study showed that an immunosuppressed environment seems to be installed early in this neoplasm, since in the operated group, in addition to CD8+ T lymphocytes, M2 macrophages also predominated.

As seen, different populations of immune cells and varied expression profiles of immune checkpoint molecules in different tumor stages can be found. This may imply different results of similar research and also varied responses to therapies.

Faced with the complex immune changes in the tumor environment of pancreatic adenocarcinoma, much more research will be necessary to unleash the body’s immune potential to overcome the important immunosuppression installed in this tumor, and thus improve the prognosis of this extremely aggressive and lethal cancer.

## Supporting information

S1 Fig Pancreatic Biopsy slide. Pancreatic Biopsy performed under Ultrasound or Computed Tomography.
(PDF)

S2 Fig Whipple surgery material slide. Representative tumor section of pancreaticoduodenectomy.
(PDF)
